# Natural killer cell-based adoptive immunotherapy eradicates and drives differentiation of chemoresistant bladder cancer stem-like cells

**DOI:** 10.1186/s12916-016-0715-2

**Published:** 2016-10-21

**Authors:** Margarida Ferreira-Teixeira, Daniela Paiva-Oliveira, Belmiro Parada, Vera Alves, Vitor Sousa, Obinna Chijioke, Christian Münz, Flávio Reis, Paulo Rodrigues-Santos, Célia Gomes

**Affiliations:** 1 Laboratory of Pharmacology and Experimental Therapeutics, Institute for Biomedical Imaging and Life Sciences (IBILI), Faculty of Medicine, University of Coimbra, Coimbra, Portugal; 2 CNC.IBILI, University of Coimbra, Coimbra, Portugal; 3 Urology and Renal Transplantation Department, Coimbra University Hospital Centre (CHUC), Coimbra, Portugal; 4 Institute of Immunology, Faculty of Medicine, University of Coimbra, Coimbra, Portugal; 5 Service of Anatomical Pathology, Coimbra University Hospital Centre (CHUC), Coimbra, Portugal; 6 Institute of Anatomical and Molecular Pathology, Faculty of Medicine, University of Coimbra, Coimbra, Portugal; 7 Viral Immunobiology, Institute of Experimental Immunology, University of Zürich, Zürich, Switzerland; 8 Center of Investigation in Environment, Genetics and Oncobiology (CIMAGO), Faculty of Medicine, University of Coimbra, Coimbra, Portugal; 9 Immunology and Oncology Laboratory, Center for Neurosciences and Cell Biology (CNC), University of Coimbra, Coimbra, Portugal

**Keywords:** Bladder cancer, Cancer stem cells, Immunotherapy, Natural killer cells

## Abstract

**Background:**

High-grade non-muscle invasive bladder cancer (NMIBC) has a high risk of recurrence and progression to muscle-invasive forms, which seems to be largely related to the presence of tumorigenic stem-like cell populations that are refractory to conventional therapies. Here, we evaluated the therapeutic potential of Natural Killer (NK) cell-based adoptive immunotherapy against chemoresistant bladder cancer stem-like cells (CSCs) in a pre-clinical relevant model, using NK cells from healthy donors and NMIBC patients.

**Methods:**

Cytokine-activated NK cells from healthy donors and from high-grade NMIBC patients were phenotypically characterized and assayed in vitro against stem-like and bulk differentiated bladder cancer cells. Stem-like cells were isolated from two bladder cancer cell lines using the sphere-forming assay. The in vivo therapeutic efficacy was evaluated in mice bearing a CSC-induced orthotopic bladder cancer. Animals were treated by intravesical instillation of interleukin-activated NK cells. Tumor response was evaluated longitudinally by non-invasive bioluminescence imaging.

**Results:**

NK cells from healthy donors upon activation with IL-2 and IL-15 kills indiscriminately both stem-like and differentiated tumor cells via stress ligand recognition. In addition to cell killing, NK cells shifted CSCs towards a more differentiated phenotype, rendering them more susceptible to cisplatin, highlighting the benefits of a possible combined therapy. On the contrary, NK cells from NMIBC patients displayed a low density on NK cytotoxicity receptors, adhesion molecules and a more immature phenotype, losing their ability to kill and drive differentiation of CSCs. The local administration, via the transurethral route, of activated NK cells from healthy donors provides an efficient tumor infiltration and a subsequent robust tumoricidal activity against bladder cancer with high selective cytolytic activity against CSCs, leading to a dramatic reduction in tumor burden from 80 % to complete remission.

**Conclusion:**

Although pre-clinical, our results strongly suggest that an immunotherapeutic strategy using allogeneic activated NK cells from healthy donors is effective and should be exploited as a complementary therapeutic strategy in high-risk NMIBC patients to prevent tumor recurrence and progression.

**Electronic supplementary material:**

The online version of this article (doi:10.1186/s12916-016-0715-2) contains supplementary material, which is available to authorized users.

## Background

Bladder cancer (BC) is the most common malignancy of the urinary tract, and one of the leading causes of cancer death in Western countries [1, 2]. Although the majority of the newly diagnosed cases are non-muscle-invasive tumors (NMIBC), up to 80 % recur and a significant part progresses to therapy refractory muscle-invasive forms (MIBC) [3, 4].

A recent report from our group demonstrated that MIBC harbor distinct cell subsets reflecting molecular features of stem-like cells endowed with enhanced chemoresistance and tumor initiating ability [5]. In addition to the inefficacy of conventional chemotherapy towards bladder cancer stem-like cells (CSCs), we also showed that a short-term exposure to cisplatin induced a phenotypic cell state transition to an adaptive stem-like phenotype, providing evidence for the tumor plasticity and spontaneous switching between cell states when subjected to stressful conditions such as chemotherapy [5]. Evidence from other groups supports our findings, reinforcing the hypothesis of a driver role of those cells in the frequent relapses of BC, as well as a fuel to the progression towards invasive forms [6, 7]. Therefore, the development of therapeutic strategies aimed to target cancer stemness is essential to prevent tumor relapse and progression, and represents an important challenge in BC management.

Natural Killer (NK) cells are important players of the innate immune system with a strong cytolytic activity against virus-infected or neoplastic cells [8] without prior immune sensitization, which make them appealing therapeutic effectors against cancer [9]. These cells secrete inflammatory cytokines and chemokines that subsequently shape the innate and adaptive immune response by promoting differentiation, activation and recruitment of accessory immune cells to the tumor site [10, 11]. The biological activity of NK cells is regulated by the dynamic balance between activating and inhibitory signals provided by the interaction with the target cells, and by soluble factors released in the tumor microenvironment, which together dictate their efficacy [12]. NK cells express a variety of activating receptors, including the NK group 2 member D (NKG2D), the DNAX accessory molecule-1 (DNAM-1), and the natural cytotoxicity receptors (NCRs: NKp30, NKp44, and NKp46), that provide activating signals upon binding to stress-induced ligands that are expressed in tumor, but not in normal cells. The inhibition of NK cells is mediated by the inhibitory killer-cell immunoglobulin-like receptors or NKG2A/CD94 that recognize classical or non-classical HLA class I molecules, respectively, which are often lost or reduced in malignant cells [13, 14]. Contrarily to conventional chemotherapy, NK cells appear to recognize and kill undifferentiated stem-like cells [15, 16] by virtue of their ability to target non-dividing cells and due to the low expression of MHC class I molecules and possible up-regulation of stress-induced activation ligands [17–19].

These findings, along with the relevance of CSCs in BC progression and the inefficacy of current therapies, prompted us to evaluate the therapeutic potential of adoptive NK cell-based immunotherapy in the eradication of competent CSCs and its impact on tumor progression, an approach that is yet to be explored in BC.

## Methods

### Cell lines

Human BC (HT-1376 and UM-UC3) and the leukemic (K562) cell lines (American Type Culture Collection, Manassas, VA, USA) were cultured in RPMI 1640 medium (Gibco, Scotland, UK) supplemented with 10 % heat inactivated fetal bovine serum (FBS), 200 mM of L-glutamine (Sigma, St. Louis, USA), and penicillin (100 IU/mL)-streptomycin (100 mg/mL) (Gibco, Scotland, UK), at 37 °C in a 5 % CO_2_ incubator. CSCs were isolated from the BC cell lines as described previously [5].

### Isolation of NK cells from healthy donors and bladder cancer patients

Polyclonal NK cells were isolated from healthy donor (HD, n = 30, mean age: 45 years old) buffy coats provided by the Portuguese Blood and Transplantation Institute or from the blood of BC patients after receiving informed consent and approval by the Institutional Review Board of Coimbra University Hospital (Approved ID: 018-CE-2016). BC patients’ blood was collected from a cohort of 10 male patients (mean age of 70 years) classified as Ta high-grade NMIBC before surgical treatment. Peripheral blood mononuclear cells were separated by density gradient centrifugation on Ficoll-Hypaque (GE Healthcare, Uppsala, Sweden). NK cells were subsequently isolated by negative selection using the NK-cell isolation kit (Miltenyi Biotec) according to the manufacturer’s instructions. Purified NK cells were cultured in complete RPMI-1640 medium (10^6^/mL) supplemented with 10 % heat inactivated FBS, 200 mM of L-glutamine (Sigma), penicillin (100 IU/mL), and streptomycin (100 mg/mL). For activation and expansion, NK cells were incubated with the interleukins IL-2 (250 IU/mL) and IL-15 (0.1 mg/mL) (Peprotech, Rocky Hill, NJ, USA) for 24 and 48 h. The purity of the isolated CD3^−^CD56^+^ NK cell populations was > 95 % in all experiments.

### Immunophenotyping of NK cells isolated from healthy donors and bladder cancer patients

NK cells were stained with fluorochrome-conjugated monoclonal antibodies against the following human surface antigens: CD56-PE-Cy7, CD16-APC-H7, CD3/CD14/CD19-PerCP-CY5.5, CD94/CD27/CD62L-FITC, NKG2C/NKp30/NKp46/NKG2D-APC, CD11b-PB, and NKG2A/NKp44/NKp80-PE (all purchased from Biolegend, San Diego, CA, USA). For intracellular staining, cells were washed, fixed, and permeabilized with Fix & Perm cell fixation and permeabilization kit (Invitrogen, Carlsbad, CA, USA) and stained with IL-4/TGF-β-FITC, TNF-α-PE, IL-10-APC, and IFN-γ-PB. Appropriate isotype controls were used. A minimum of 100,000 events were acquired using a FACSCanto II flow cytometer (BD Biosciences, San Jose, CA, USA) and analyzed with the FlowJo analysis software (Tree Star, Inc., Ashland, USA). Results were expressed as the percentage of positively stained cells in the NK cell gate.

### Immunophenotyping of BC cells

Single-cell suspensions of parental and corresponding sphere-forming cells were stained for 30 min at 4 °C with fluorescent conjugated monoclonal antibodies against HLA-ABC (clone w6/32, BioLegend), MICA/B (clone 6D4, BioLegend), ULBP1 (clone 170818, R&D Systems, Minneapolis, MN, USA), CD48 (clone 394607, R&D Systems), Nectin-2/CD112 (clone 610603, R&D Systems), CD155/PVR (clone 300907, R&D Systems), and Fas/CD95 (clone 2R2, eBiosciences, San Jose, CA, USA). For experiments with the supernatant of NK cells (NK-SN), spheres were previously incubated for 4 h with the supernatants of IL-2- and IL-15-activated NK cells before phenotyping. Appropriate isotype-matched controls were run with each experiment. Samples were analyzed using a FACSCanto II cytometer. A minimum of 100,000 events were collected and analyzed using the FlowJo software.

### CD107a degranulation and cytokine production

Freshly and IL-2/IL-15-activated NK cells (10^6^ cells) collected from HDs were co-cultured with target cells at an effector-to-target (E:T) ratio of 3:1 in U-bottomed 96-well plates for 4 h in a 5 % CO_2_ incubator with PE-conjugated anti-CD107a (H4A3, BioLegend) and Brefeldin A (Golgistop, BD). Stimulus with 25 ng/mL PMA plus 250 ng/mL ionomycin was used as a positive control and NK cells alone were used as a negative control. Cultured cells were then stained with fluorochrome-conjugated monoclonal antibodies against human blood surface antigens: CD3 PerCP/Cy5.5 (clone HIT3a), CD14 PerCP/Cy5.5 (clone M5E2), CD19 PerCP/Cy5.5 (clone HIB19), CD16 FITC (clone 3G8), and CD56 APC (clone HCD56), all purchased from BioLegend. The percentage of CD3^−^CD56^+^ NK cells positive for CD107a was calculated. All analyses were performed in duplicate using BD FACSCanto II and FlowJo analysis software.

Cytokines produced by 48 h IL-2/IL-15-activated NK cells co-cultured with tumor cells at an E:T ratio of 10:1 were measured using ELISA kits according to the manufacturer’s instructions (granzyme B and IFN-γ: Abcam, Cambridge, UK; and TNF-α: R&D Systems, MN, USA).

### Chromium-51 (^51^Cr)-release assay

Target cells were loaded for 1 h with 50 μCi of ^51^Cr (PerkinElmer, Massachusetts), washed twice and incubated with fresh or activated NK cells at different E:T ratios (1:1, 3:1 and 10:1) in 200 μL of complete RPMI in 96-well U-bottom tissue culture plates at 37 °C in a 5 % CO_2_.

After a 4-h incubation period, the supernatants were harvested and counted for released radioactivity in a gamma counter (CRC-55tW Capintec), within a ^51^Cr sensitivity energy window (300–400 keV). The specific lysis of target cells was calculated as follows: Percentage of specific lysis = (experimental release – spontaneous release)/(maximum release – spontaneous release) × 100. Spontaneous release was calculated from target cells without effector cells. Maximum release was determined by incubating target cells with 4 % SDS detergent. In all experiments, the spontaneous release was < 20 % of maximum release.

For NK cells blocking receptor experiments, activated NK cells were pre-incubated with 10 μg/mL of anti-NKG2D (clone 149810, R&D Systems), 10 μg/mL of anti-DNAM-1 (clone 102511, R&D Systems), and 0.5 μg/mL of anti-FasL (clone ZB4, Merck Millipore, Germany), individually or in combination, before co-culture with tumor target cells.

### NK cell supernatant assays

Both parental and CSCs were cultured for 4 h with the supernatant harvested from 48-h IL-2/IL-15-activated NK cells from HDs or BC patients. Thereafter, tumor cells were assayed for aldehyde dehydrogenase (ALDH) activity, expression of stemness-related markers and cell surface ligands for NK receptors and chemosensitivity to cisplatin.

### Aldefluor assay

The activity of ALDH in tumor cells was measured using the Aldefluor kit (Stem Cell Technologies, Vancouver, BC, USA), according to the manufacturer’s instructions. FACS was performed on a BD FACSCanto II flow cytometer. Data was analyzed with the FlowJo software.

### Gene expression by real-time quantitative PCR analysis (RT-qPCR)

Total RNA from sphere-forming and parental cells was extracted using the ReliaPrep RNA Cell Miniprep System (Promega) following the manufacturer’s instructions. The quantity and quality of isolated RNA was measured by the ND-1000 spectrophotometer (NanoDrop Technologies). Reverse transcription from 1 μg of total RNA was performed using NZY First-Strand cDNA Synthesis kit (Nzytech) and subsequent RT-qPCR for SOX2, ABCG2, ABCB1, ALDH1A1, ALDH2, CD44, CD47, and KRT14 was performed as previously described [5]. Primers used on RT-qPCR reaction are listed in Additional file 1: Table S1. mRNA expression was normalized to three housekeeping genes: *18S*, *GAPDH*, and *HRPT-1* using the ΔΔCt method and Bio-Rad CFX Manager™ 3.0 software.

### Chemosensitivity to cisplatin

Cells were treated with increasing concentrations of cisplatin (Teva Pharma, Portugal) ranging from 1 to 100 μM over 48 h. Cell viability was analyzed using the standard MTT [3-(4,5-dimethylthiazol-2-yl)-2,5-diphenyltetrazolium bromide] (Sigma) assay as previously described [5]. Cell viability was expressed as the percentage of absorbance values of the treated cells related to the untreated control wells considered as 100 %.

### Bladder tumor specimens and immunohistochemistry

Bladder tumor samples were obtained from 25 patients (19 males and 6 females) by transurethral resection at Coimbra University Hospital, following appropriate informed consent and ethical regulatory approval (Approved ID: 018-CE-2016). Tumors at initial diagnosis were stratified into non-muscle-invasive low (n = 15) and high (n = 7) grade and muscle-invasive tumors (n = 3) by a pathologist, according to the 2004 WHO criteria [20]. Formalin-fixed paraffin-embedded tissue blocks were sectioned at 3-μm thickness and incubated in a BenchMark Ultra Ventana, with a primary antibody against CD56, a surface marker for NK cells, clone 123C3 (1:50, Roche), for 30 min at 37 °C, and reaction signal was developed with 3-3′-diaminobenzidine tetrahydrochloride chromogen. Standard procedures were used for visualization and the intensity and percentage of positive staining was registered. Two investigators blinded to the data reviewed all slides independently.

### Animal studies

Animal studies were approved by the Organization Responsible for Animal Welfare of the Faculty of Medicine of Coimbra (Approved ID: ORBEA/91/2015/08) and were performed according to National and International guidelines on animal experimentation. Female nude mice (Swiss nu/nu), 6–8 weeks old (Charles River Laboratories, Barcelona, Spain) were housed under pathogen-free conditions in individual ventilated cages. The subcutaneous tumor model was induced by subcutaneous injection into the lower flank of 1 × 10^6^ of Luc^+^ HT-1376 cells suspended in 100 μL of a 1:1 PBS/Matrigel mixture. The orthotopic model that more closely resembles the clinical and histopathological features of primary MIBC was developed by intravesical instillation of Luc^+^ HT-1376 cells as previously described [5]. Bioluminescent images were taken 24 h post-implantation and every 3 days to monitor engraftment and growth of tumor cells using an IVIS Lumina XR (Caliper Life-Sciences, Hopkinton, MA, USA) after intraperitoneal injection with D-luciferin (150 mg/kg, Synchem, BHg, Germany) with the animals under anesthesia (100 mg/kg ketamine and 2.5 % of chlorpromazine solution). Quantification of bioluminescent signals was performed using the living image software version 4.10 (Xenogen). Values are expressed as photons/sec/cm^2^/sr. Subcutaneous tumors started the treatment on day 6 post-implantation by intratumoral inoculation of NK cells activated for 48 h (5 × 10^6^/50 μL) from HDs twice a week over 2 weeks.

Animals bearing subcutaneous or orthotopic tumors were treated twice a week with healthy 48-h activated-NK cells (5 × 10^6^/mouse) via intratumoral and intravesical instillation, respectively, over 2 weeks. NK cells were washed prior to administration and resuspended in PBS. Tumor progression was monitored by bioluminescent images 3 days after each treatment. Animals were sacrificed after treatments or when presenting hematuria or lost 20 % of initial body weight. Residual tumors were excised and processed into paraffin blocks for immunohistochemistry analysis of CD56 clone 123C3 (1:50, Roche) and for two CSC-related markers, SOX-2 (clone D6D9, 1:100, Cell Signaling) and ALDH2 (clone EPR4493, 1:100, Abcam) as described above for clinical samples.

### Statistical analysis

Data are reported as the means ± SEM of the indicated number of experiments. Statistical analysis and graphic illustrations were performed using GraphPad Prism 6.0 software (San Diego, CA). Paired two-tailed Student’s t-tests, ANOVA, and Tukey’s tests were used to calculate *P* values. A *P* value of less than 0.05 was considered significant.

## Results

### Activated-NK cells from healthy donors are highly effective against chemoresistant bladder cancer stem-like cells

**Fig. 1:**
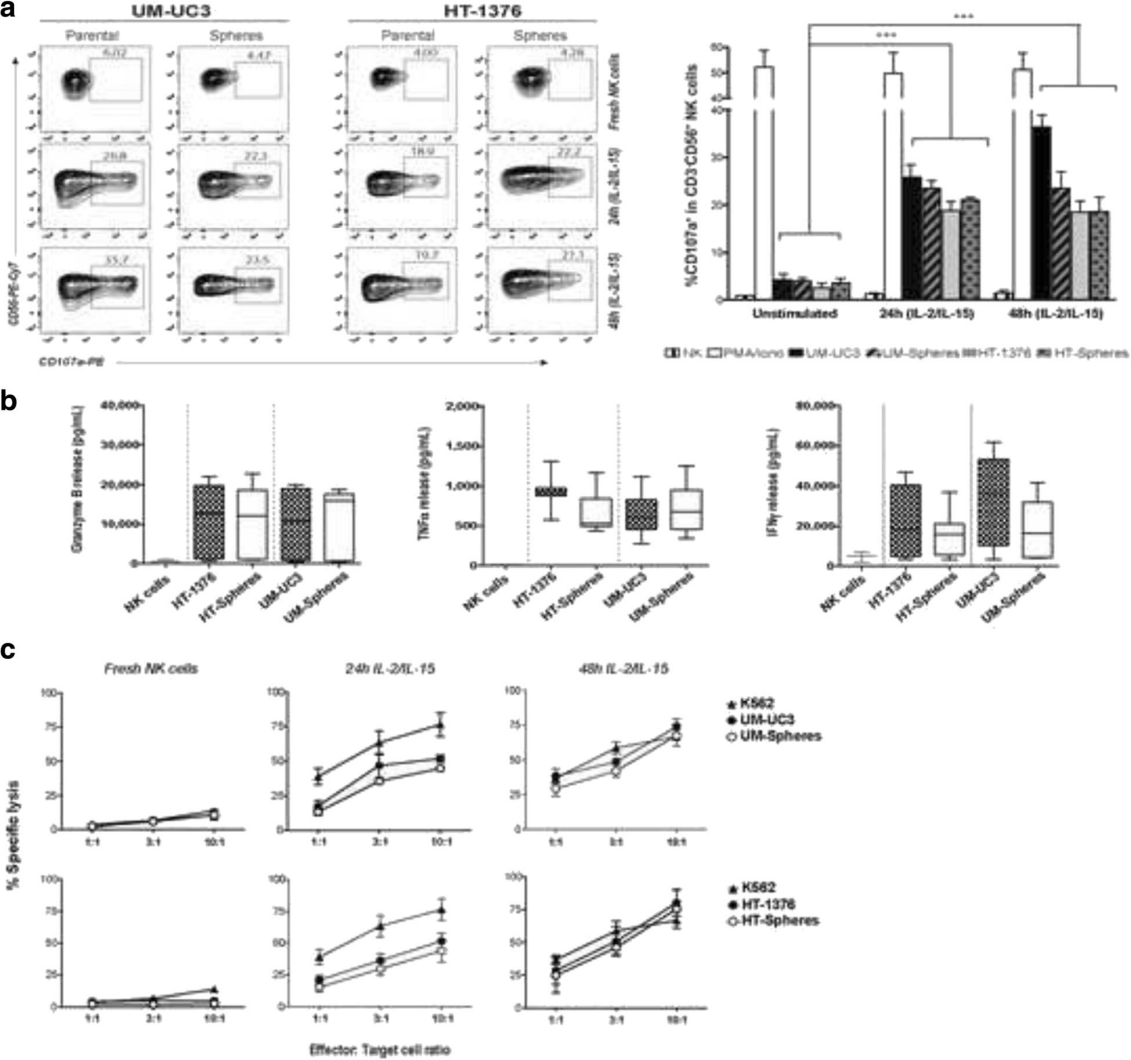
Allogeneic activated-NK cells are effective against bladder cancer stem and non-stem cells. **a** Representative FACS dot-plots of CD107a release in resting and 24- or 48-h IL-2/IL-15-activated CD3^−^CD56^+^ NK cells after 4 h co-culture with spheres and corresponding parental cells at a 3:1 effector-to-target (E:T) ratio. The graph shows the percentage of CD3^−^CD56^+^CD107a^+^ expressing cells. PMA/ionomycin was used as the positive control and NK cells alone as the negative control. **b** Release of IFN-γ, TNF-α, and granzyme B by 48-h activated NK cells after 4 h co-culture with parental or spheres at an E:T ratio of 10:1, determined by ELISA. Graphs represent the means ± SEM, n = 4. **c** Percentage of dead target cells measured by the ^51^Cr-release assay after 4 h co-culture with resting and IL-2/IL-15-activated NK cells at different E:T ratios. K562 cells were used as a positive control. Graphs show the mean ± SEM, n = 5

The functional activity of NK cells from HDs against parental cells and CSCs was evaluated by measuring CD107a degranulation, release of cytokines, and lysis of target cells following a 4 h co-culture period. Freshly isolated NK cells were in the resting state in all E:T ratios tested, as indicated by the low CD107a degranulation rates, displaying weakly cytolytic activity against any cell line including the MHC class I-negative K562 cells. Upon stimulation with IL-2/IL-15, NK cells enhanced their functionality and cytotoxicity against either parental cells or CSCs, as demonstrated by the enhanced CD107a degranulation rates, and release of IFN-γ, TNF-α, and granzyme B (a lytic granule) as compared to fresh NK cells (Fig. 1a, b). During the 24 and 48 h of activation, the percentage of viable NK cells decreased to 20–30 %.

The cytolytic activity of NK cells, measured by the ^51^Cr-release assay, increased with increasing E:T ratio and reached a specific lysis greater than 70 % for an E:T ratio of 10:1 in both cell subsets upon 48 h activation with IL-2/IL-15 (Fig. 1c). No significant differences were obtained between CSCs and parental cells, indicating equal susceptibility of BC cells to activated NK cells lysis.

Flow cytometry analysis of the various receptors involved in NK cell effector functions showed a significant up-regulation of the NCRs NKp44 (2.00 ± 1.16 % vs. 26.33 ± 3.84 %, *P* < 0.01) and NKp30 (0.12 ± 0.02 % vs. 2.68 ± 0.33 %, *P* < 0.01), and of the NKG2D (65.00 ± 9.45 % vs. 96.33 ± 1.76 %) and DNAM-1 (78.67 ± 3.66 % vs. 92.25 ± 1.65 %) activating receptors upon 48 h activation, relative to resting NK cells (Additional file 2: Figure S1), indicating the crucial role of stimulatory cytokines in NK cell antitumor properties.

Moreover, the distribution of gated CD56^+^CD3^−^ NK cells with regard to CD16 expression changed upon stimulation with IL-2/IL-15, resulting in a significant increase in the CD16^−^ subpopulation in relation to resting NK cells. The median percentage of CD56^bright^CD16^−^, which in freshly NK cells was of 2.68 ± 0.20 % (2.34–3.29 %), increased to 4.32 ± 0.21 % (3.98–4.85 %) and to 8.57 ± 1.02 % (6.64–10.10 %) upon 24- and 48-h cytokine activation, respectively. No significant changes were observed in the percentage of CD56^dim^CD16^+^ cells.

### Bladder CSCs display increased expression of ligands recognized by NK cell activating receptors

**Fig. 2:**
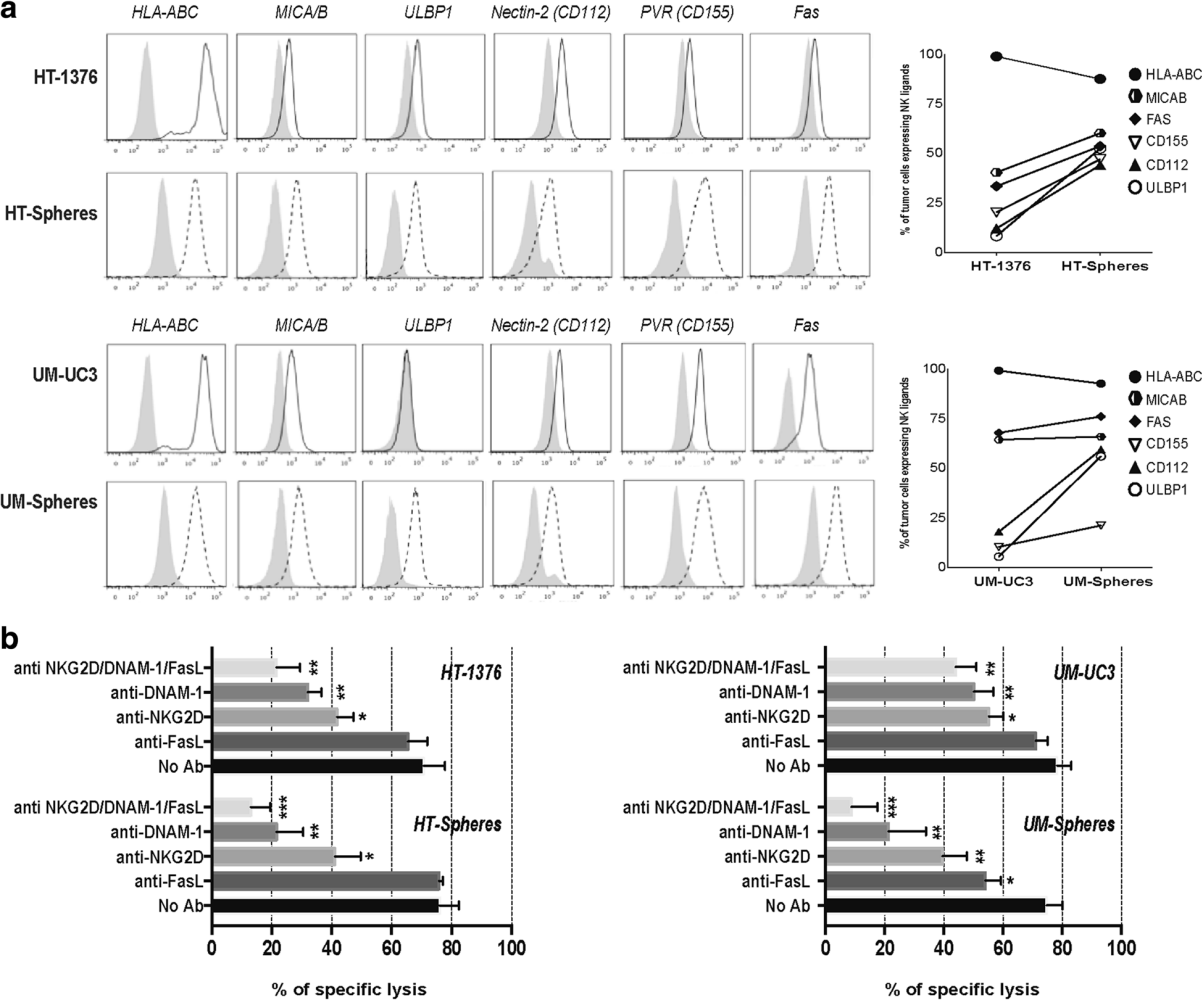
Bladder cancer cells express multiple ligands for NK-cell activating receptors. **a** Representative histograms illustrating the expression of HLA-ABC, CD112, CD155, MICA/B, ULBP-1, and Fas in parental cells (black solid lines) and spheres (black dotted lines). Gray profiles represent isotype-matched controls. The graph represents the mean percentage of each ligand in both cell subsets of three independent experiments. **b** Percent killing of target cells after 4 h co-culture with NK cells activated for 48 h in the presence of blocking antibodies against NKG2D, DNAM-1, and FasL, separately and in combination. Bar graph represents the mean + SEM (n = 4). *P < 0.05, **P < 0.01, and ***P < 0.001 compared to untreated NK cells

To evaluate the ability of BC cells to stimulate NK-mediated cytotoxicity, both parental cells and CSCs were characterized regarding the expression of ligands that engage activating and inhibitory NK receptors. Both parental cells and CSCs expressed activating ligands involved in NK recognition, namely MICA/B and ULBP-1 ligands for NKG2D-activating receptor and PVR and Nectin-2 for DNAM-1, as well as the Fas death receptor (Fig. 2a). Interestingly, all activating ligands were found more highly expressed in the CSC subsets in comparison to corresponding parental cells. The HLA-class I molecules (HLA-ABC), which play a major role in NK cell inhibition, were expressed in both BC cell lines and slightly decreased in spheres.

### NKG2D and DNAM-1 activating receptors mediate bladder CSC lysis

To identify the contribution of the different activating receptors behind NK cell recognition of target cells, we performed blocking studies using specific monoclonal antibodies. As indicated by the killing assay (Fig. 2b), blocking NKG2D (*P* < 0.05) and DNAM-1 (*P* < 0.01) receptors impaired the overall cytolytic activity of NK cells against both BC cell subsets. Additionally, Fas-L blocking decreased the ability of NK cells to kill the stem-like fraction of the UM-UC3 cell line in agreement with the high surface expression levels of Fas in these cells. The combined mAb-mediated blocking of NKG2D, DNAM-1, and Fas-L receptors almost completely abrogated NK cell-mediated killing of spheres from the two BC cell lines, in agreement with the higher density of ligands interacting with these specific NK-activating receptors.

### Supernatants from NK cells induce differentiation and sensitize CSCs to cisplatin

In addition to increased chemoresistance, CSCs are characterized by their ability to self-renew and differentiate. We tested whether NK cells could induce CSCs towards a more differentiated phenotype rendering them susceptible to chemotherapy.

**Fig. 3:**
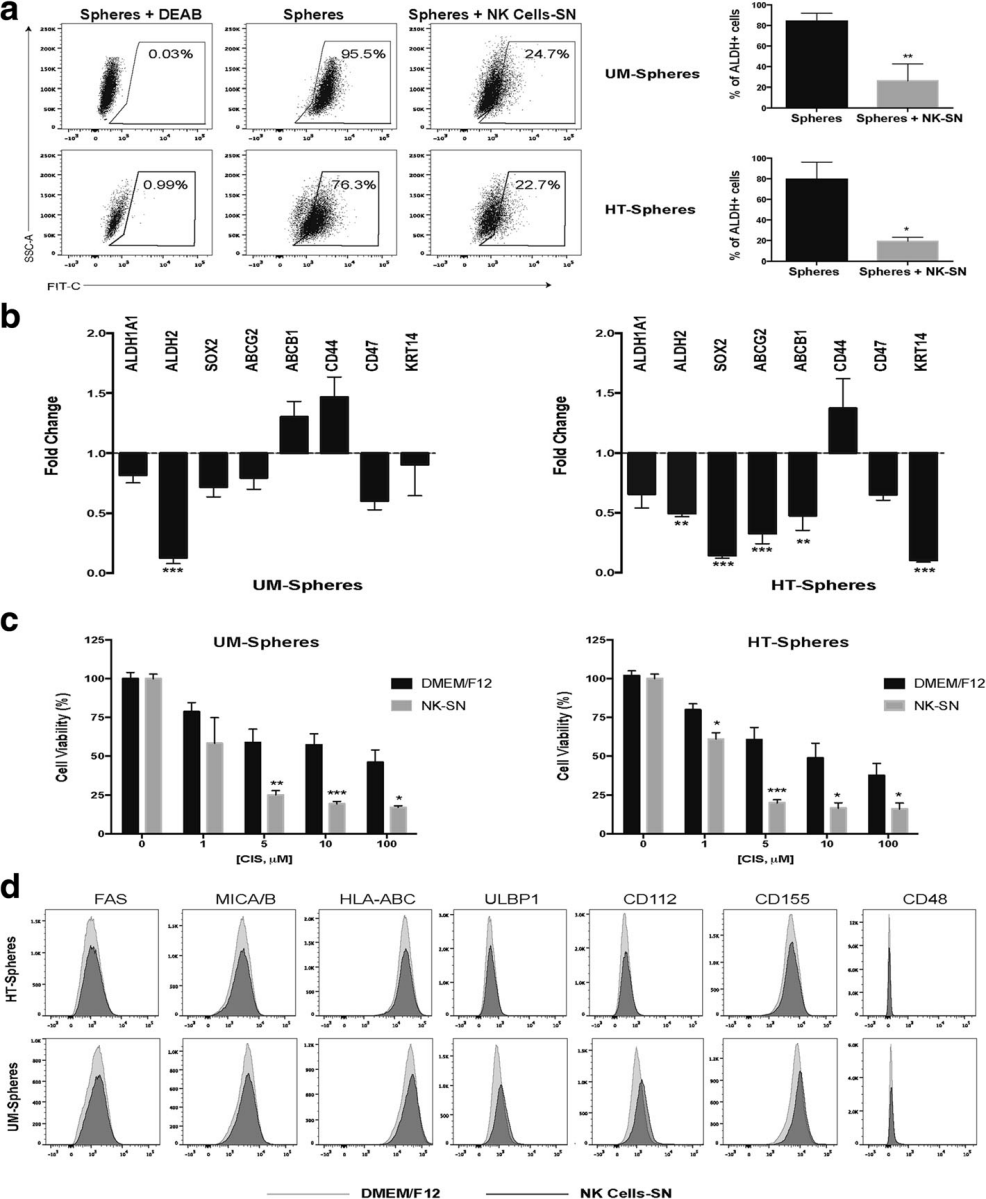
NK cell-derived supernatant (SN) promotes the differentiation of cancer stem-like cells and increases their sensitivity to cisplatin. **a** Representative flow cytometry analysis of ALDH enzymatic activity in spheres after 4 h with NK-derived SN. The ALDH inhibitor DEAB was used as a negative control to establish the baseline fluorescence of the cells. Bar graph shows the percentage of ALDH^+^ cells (mean ± SEM, n = 3). **b** Fold-change mRNA expression levels of stemness-related markers in spheres treated with NK-SN relative to untreated spheres set as 1 (mean ± SEM, n = 5). **c** Cytotoxic effects of cisplatin in spheres cultured in DMEM/F12 (black bars) and upon culture with NK-SN (gray bars) for 4 h determined by the MTT assay. The percentage of viable cells was normalized respective to untreated cells (mean ± SEM, n = 3). **d** Representative histograms illustrating the expression of specific ligands on the surface of spheres cultured in DMEM/F12 medium (gray histograms) or previously incubated with NK-SN for 4 h (black histograms). *P < 0.05, **P < 0.01, and ***P < 0.001 represents spheres in NK-SN vs. spheres in regular DMEM/F12

Therefore, spheres were incubated with the supernatants of activated-NK cells for 4 h, followed by analysis of stemness related markers previously identified [5]. The ALDH activity, considered a functional readout of stemness, decreased by 60 % in spheres after 4 h incubation with NK supernatants (Fig. 3a). Accordingly, the transcript levels of two ALDH isoforms responsible for ALDH activity (ALDH1A1 and ALDH2) were also down regulated in both CSC populations (Fig. 3b). The mRNA expression levels of other stem cell-related markers, including pluripotency factors (SOX2, POU5F1, and NANOG), urothelial basal cell-specific markers (CD44, CD47, and KRT14), and drug resistance-related transporters (ABCG2 and ABCB1), were also significantly downregulated in HT-1376 spheres. A similar trend, although not significant, was noticed in UM-UC3 spheres. No significant transcription changes were observed in corresponding parental cells (data not shown). Additionally, pre-treatment with the NK cell supernatant sensitized CSCs towards cisplatin, a drug currently used in the treatment of MIBC, as compared to non-pretreated cells (Fig. 3c).

### NK cells from bladder cancer patients display low expression of NCRs and fail to mediate CSCs lysis

**Fig. 4:**
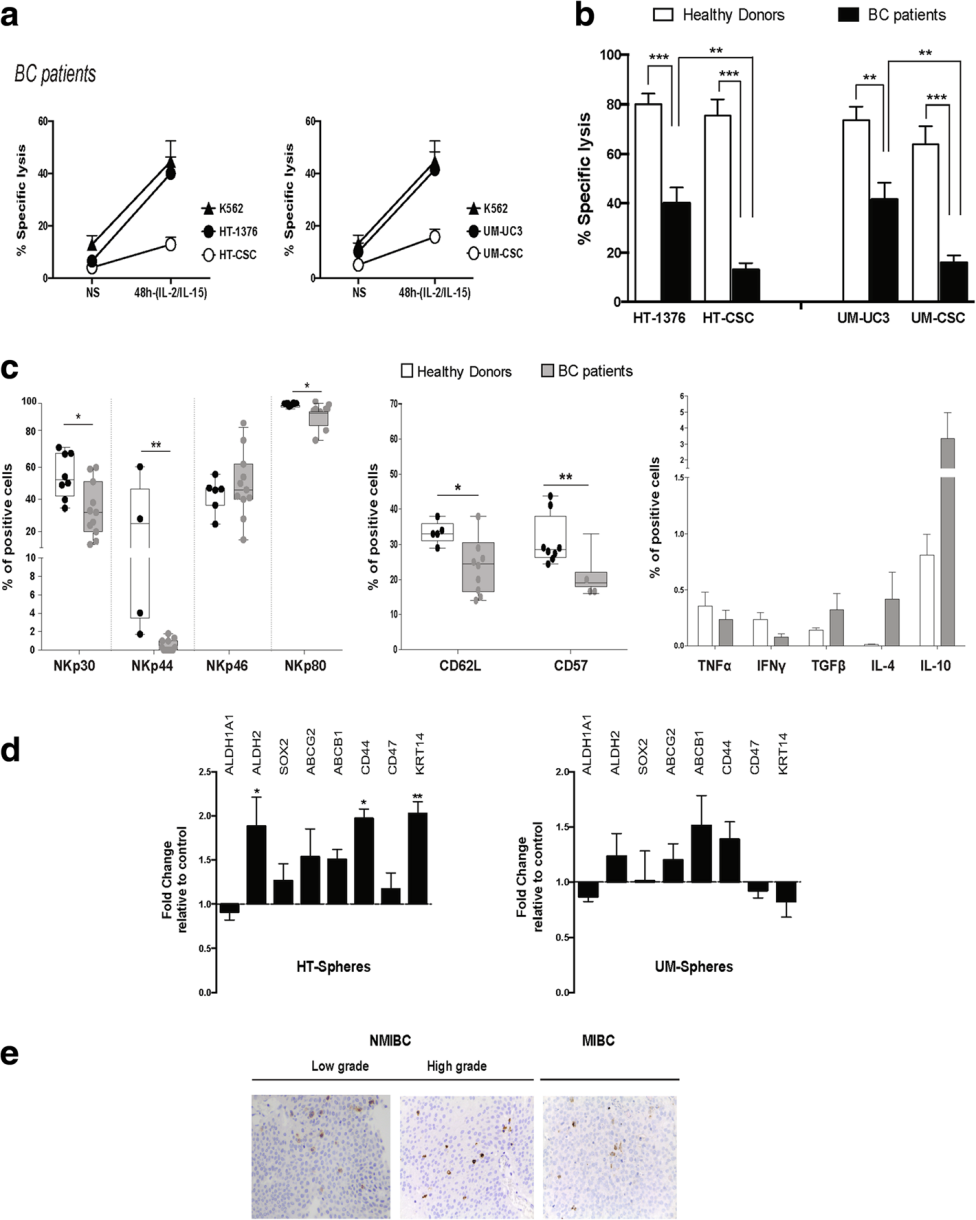
NK cells from bladder cancer (BC) patients have impaired cytolytic activity and are ineffective against cancer stem-like cells. **a** Cytolytic activity of resting and IL-2/IL-15-activated NK cells after 48 h of activation derived from BC patients against BC parental cells and spheres after 4 h co-culture at a 10:1 ratio measured by the ^51^Cr release assay. **b** Comparative analysis of lytic activity of NK cells derived from HDs and BC patients against spheres and parental cells. **c** Flow cytometry analysis of the natural cytotoxicity receptors, adhesion molecule CD62L, differentiation marker CD57, and the cytokine profile of BC patient (gray boxes, n = 10) and HD (white boxes, n = 8) NK cells. Graphs represent the percentage of positive cells (mean ± SEM). *P < 0.05, **P < 0.01, and ***P < 0.001 HD versus BC patients. **d** Fold-change mRNA expression levels of stemness-related markers in spheres treated with NK-SN from BC patients relative to untreated spheres set as 1 (mean ± SEM, n = 5, *P < 0.0 5 and **P < 0.01). **e** Immunohistochemical staining for CD56 in serial sections of tumoral tissue from BC patients with non-muscle-invasive, low grade; non-muscle-invasive, high grade; and muscle-invasive tumors. Original magnification: ×400

Next, we analyzed the phenotypic status and functionality of NK cells collected from the peripheral blood of high-grade NMIBC patients with high risk of recurrence. NK cells displayed decreased responsiveness to cytokine stimulation, as indicated by the overall lower specific lysis observed in both cell subsets comparatively to activated-NK cells from HDs (Fig. 4a, b), with considerably reduced cytotoxicity against spheres (*P* < 0.01), contrary to healthy NK cells, which displayed an equal capacity to kill stem and parental cells (Table 1). Phenotypic analysis showed a reduced expression of NKp30, NKp44, and the co-receptor NKp80 in patient NK cells, as compared with HDs (Fig. 4c). The expression of the adhesion molecule CD62L and the terminal differentiation marker CD57 was significantly decreased in NK cells from BC patients. Furthermore, NK cells from patients showed up-regulation of the immunosuppressive anti-inflammatory cytokines TGF-β, IL-4, and IL-10, and down regulation of pro-inflammatory cytokines TNF-α and IFN-γ, in agreement with the impaired NK cell activity (Table 1).

**Table 1:** Profile of NK cells derived from healthy donors and bladder cancer patients

	Healthy donors	Bladder cancer patients
^51^Cr-release assay		
*HT-1376*	80.12 ± 4.14 %	38.67 ± 8.88 %***
*HT-1376 spheres*	75.44 ± 6.53 %	11.74 ± 3.12 %***
*UM-UC3*	73.69 ± 5.40 %	41.38 ± 10.71 %**
*UM-UC3 spheres*	67.52 ± 7.61 %	18.19 ± 4.17 %***
NCRs		
*NKp30*	54.68 ± 4.98 %	33.55 ± 5.09 %*
*NKp44*	26.35 ± 9.18 %	0.52 ± 0.20 %***
*NKp46*	42.93 ± 3.70 %	49.09 ± 6.09 %
*NKp80*	98.34 ± 0.32 %	91.36 ± 2.20 %**
CD62L	33.40 ± 1.44 %	24.30 ± 2.57 %*
CD57	31.08 ± 2.54 %	20.86 ± 2.14 %**
Cytokines		
*TNF-*α	0.36 ± 0.13 %	0.23 ± 0.08 %
*IFN-*γ	0.23 ± 0.06 %	0.08 ± 0.03 %
*TGF-β*	0.14 ± 0.02 %	0.32 ± 0.15 %
*IL-4*	0.007 ± 0.003 %	0.42 ± 0.24 %
*IL-10*	0.81 ± 0.19 %	3.36 ± 1.62 %

^*51*^ *Cr* chromium-51, *NCRs* natural cytotoxic receptors

*P < 0.05; **P < 0.01; ***P < 0.001 healthy donors (n = 8) vs. bladder cancer patients (n = 10)

Moreover, exposure of CSCs to NK supernatants derived from BC patient cells did not decrease the expression of stemness-related markers in spheres. Rather, a trend was observed towards up-regulation of the majority of analyzed genes, suggesting that NK cells release factors that maintain or exacerbate the stemness features of tumor cells (Fig. 4d).

To further evaluate whether tumor-infiltrating NK cells might indeed represent an ongoing anti-immunity response in BC, we analyzed the expression of CD56^+^ NK cells in a panel of human BC samples classified as low- and high-grade NMIBC and MIBC at diagnosis. Our results revealed a small percentage of infiltrating CD56^+^NK cells within tumors in all tumor stages, indicating these tumors are not infiltrated by NK cells, being unlikely to greatly contribute to the elimination of tumor cells (Fig. 4e).

### Adoptive transfer of healthy activated-NK cells display anti-tumor activity in bladder cancer xenografted models

Given the considerable low cytotoxic activity of NK cells from BC patients, we focused on the anti-tumoral activity of NK cells from HD in animal models induced by xenotransplantation of HT-1376 cells. The HT-1376 cell line contains a subpopulation of CSCs, as previously demonstrated by the presence of an ALDH^+^ population with sphere-forming ability, and forms an orthotopic heterogeneous tumor resembling the clinical condition of MIBC comprising stem-like and proliferative differentiated cell populations, as previously demonstrated by our group [5].

**Fig. 5:**
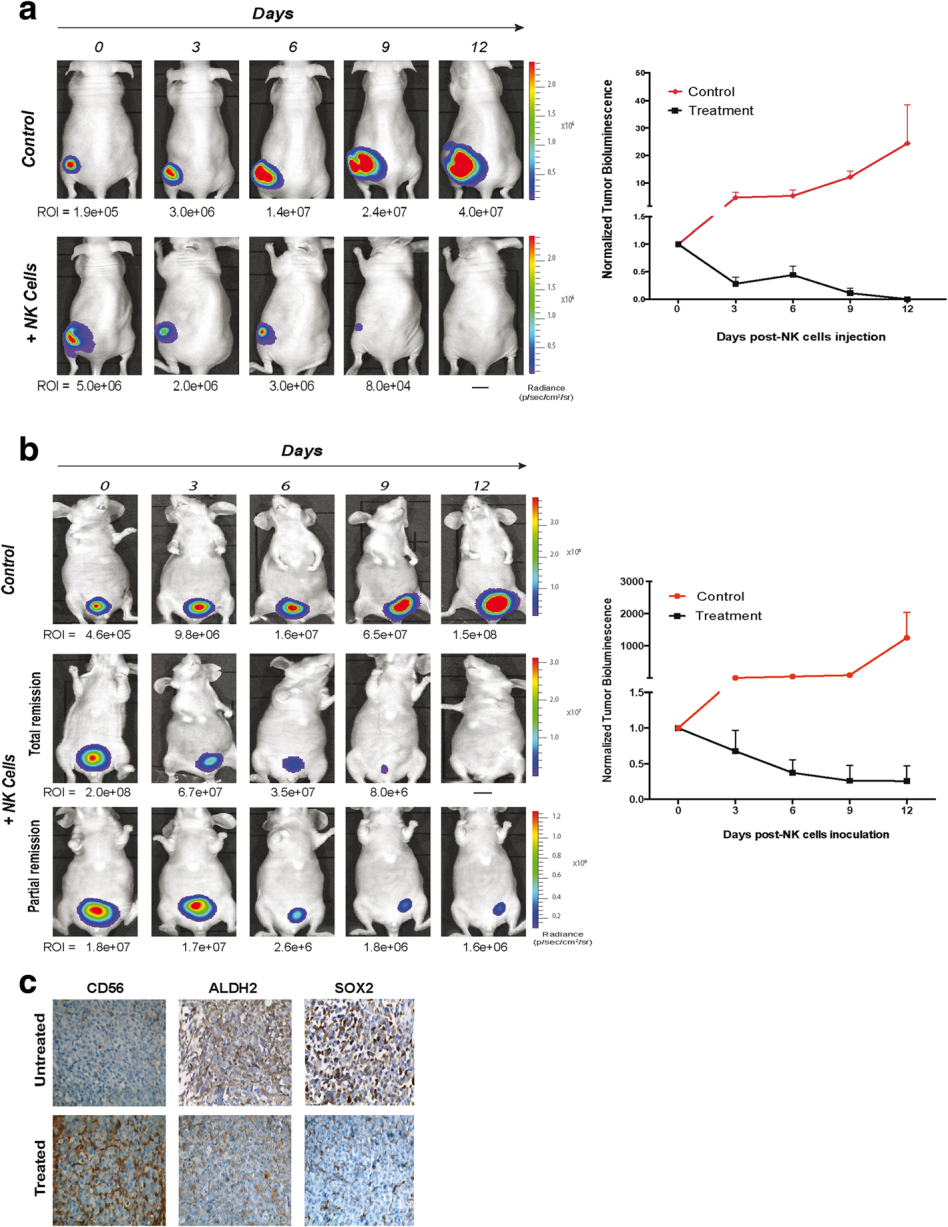
Allogeneic activated-NK cells from healthy donors are effective in eradicating cancer stem-like cells in bladder cancer mouse models. **a** Serial bioluminescent images of a representative subcutaneous tumor-bearing mouse after intratumoral injection of activated-NK cells. **b** Anti-tumoral effects of activated-NK cells administered intravesically in mice bearing an orthotopic bladder cancer. All animals were treated twice a week with 5 × 10^6^ NK cells for 2 weeks and were monitored each 3 days after treatment. The tumor size was evaluated by quantification of the bioluminescent signal (photons/sec/cm^2^/sr) in a region of interest drawn around the tumor. The graphs represent tumor progression of untreated and treated animals normalized to the bioluminescent signal at the beginning of the treatment (n = 5, per group). **c** Immunohistochemical staining for CD56, ALDH2 and SOX2 in serial sections of an orthotopic untreated tumor (upper row) and in a residual treated tumor after the fourth intravesical administration of activated-NK cells (lower row). Original magnification: ×400

First, we evaluated the antitumor activity of NK cells in mice bearing localized subcutaneous tumors. The treatment started 6 days after cell inoculation and was performed twice a week by intratumoral injection of 5 × 10^6^ activated-NK cells. An immediate and progressive decrease in the tumor size was observed, being totally abolished after the fourth administration (Fig. 5a). At that time, the treatment was finished and the animals were monitored for up to 2 weeks, and no tumor relapse was observed. Thereafter, we tested the same approach, but in an organ-specific microenvironment using an orthotopic model (Fig. 5b). NK cells were instilled intravesically into the bladder lumen 4 weeks after tumor cells implantation. The treatment resulted in a progressive decrease of tumor burden by 80 % after the fourth inoculation with total remission in one of the five animals treated.

The immunostaining of residual tumors showed a high degree of CD56^+^ tumor-infiltrating NK cells, and a marked reduction of two stemness markers (SOX-2/ALDH2) expression in treated tumors, compared to untreated controls (Fig. 5c), confirming the CSC-targeting ability of locally administered NK cells in an organ-specific microenvironment.

## Discussion

A major challenge in BC treatment is the risk of progression to muscle-invasive forms or metastatic disease, a process that appears to be strongly related to the presence of CSCs that are resilient to current conventional therapies. We provided evidence that both stem and non-stem cells can be recognized and effectively killed by ex vivo-activated allogeneic NK cells from HDs, but not from BC patients.

Compared with resting cells, cytokine-activated NK cells displayed an increased density of major activating receptors, crucial for NK cell cytokine production and cytotoxicity. Moreover, the expansion of CD56^bright^CD16^−^ NK cells is likely to contribute to the enhanced NK cell-mediated cytotoxicity. Although the lytic activity of NK cells is generally attributed to the CD56^dim^ subset, it is conceivable that the CD56^bright^ subset becomes more mature and equally cytotoxic as the CD56^dim^ subset following cytokine stimulation. In line with this, Romee et al. [21] showed that IL-15 primed CD56^bright^ NK cells with a highly potent antitumor activity in acute myeloid leukemia.

The phenotypic analysis of BC cells confirmed the high expression levels of several ligands recognized by NK activating receptors in both tumor cell subsets, making them suitable targets for NK cell-based immunotherapy. The levels of HLA class I molecules were not enough to protect tumor cells from NK-mediated lysis, suggesting that the susceptibility of BC cells might not entirely depend on missing self-recognition and that NK cell activation induced by activating ligands is a strong mechanism to overcome MHC class I inhibitory signals. This is consistent with a previous report showing that the loss of MHC inhibitory signals did not change the pattern of NK cell degranulation towards BC cells [22].

The decreased lytic activity observed in receptor blocking experiments confirmed the importance and cooperation pattern of DNAM-1- and NKG2D-dependent mechanisms in trigger activation signals and in overcoming the inhibitory signals resulting from MHC-I recognition, in both cell subsets. We cannot exclude that this alloreactivity of NK cells against tumor cells might also be caused by a killer-cell immunoglobulin-like receptor/HLA receptor-ligand mismatch, an aspect that was not explored in this work and deserves investigation.

Importantly, NK cells release critical factors that regulate the switch of spheres into a more differentiated status, thus reversing their resistance to cisplatin and indicating a dual effect on depletion of the CSC pool by direct killing and by generation of differentiated cells vulnerable to conventional therapies. This inducing-differentiation effect, already described and referred to as split energy, has been attributed to anergized NK cells that lose cytotoxicity but augmented the secretion of cytokine (IFN-γ, TNF-α) inducers of CSC differentiation [19, 23].

NK cells from BC patients are less responsive to cytokine activation and display a reduced lytic activity, especially against CSCs, due to the low expression of NCRs and CD62L, crucial for recognition and killing of target cells, and by the presence of immature CD57^−^ NK cells. Apart from the modified NK phenotype, the increased levels of immunosuppressive cytokines (TGF-β, IL-10, and IL-4) and reduced expression of IFN-γ/TNF-α prevented an effective antitumor immune response and abolished their differentiation-inducing effects on CSCs [24, 25]_._ Finally, factors released by NK cells from BC patients were ineffective in driving differentiation of CSCs, which may contribute to the expansion of the CSC pool and subsequent tumor progression.

Recent studies suggest that malignant cells can bypass NK surveillance by releasing soluble forms of the NKG2D ligands, suppressing NK cell-mediated cytotoxicity. Marked levels of the soluble form of the MHC class I-related chain A, identified as a human NKG2D ligand, were found in the sera of patients with disseminated head-and-neck squamous cell [26] and human hepatocellular carcinomas [27] and neuroblastoma [28]. This tumor-derived soluble inhibitory ligand appears to be responsible for the downregulation of NKG2D expression in NK cells and subsequent impaired NKG2D-mediated cytotoxicity in patients with advanced disease. This mechanism also impairs the adaptive immunity due to the loss of allostimulatory capacity of dendritic cells mediated by NK cells [27]. Moreover, the downregulation of NKG2D was also reported in infiltrating and matched peripheral blood T cells in cancer patients with circulating tumor-derived soluble MHC class I-related chain A, suggesting this ligand can induce an impairment of the responsiveness of tumor antigen-specific effector T cells, leading to tumor escape from immunosurveillance [29]. The existence and nature of this immunosuppressive mechanism should be further explored in BC patients.

These findings highlight the role of the tumor microenvironment in host immune response impairment and NK lytic function, and may explain the poor efficacy of adoptive transfer of autologous NK cells frequently observed in cancer patients with melanoma, lymphoma, and breast cancer due to NK-cell dysfunction [30–32]. Immunohistochemical analysis of CD56^+^ infiltrating-NK cells in biopsy specimens of BC patients revealed a poor tumor infiltration, independently of tumor stage and grade, reflecting an inefficient homing of NK cells in BC, unlikely to control tumor progression. Similar results were reported by Kripna et al. [33] in BC samples, suggesting tumor-infiltrating NK cells are not a prognostic factor in BC, contrarily to other tumor types.

The in vivo studies showed a remarkable anti-tumor activity of healthy activated-NK cells in BC xenografted models. The intratumoral delivery of activated-NK cells leads to a complete abolishment of subcutaneous tumors with no evidence of recurrence, likely reflecting the ability of NK cells to kill CSCs and non-CSCs. In the orthotopic model, the intravesical administration of NK cells resulted in a massive decrease in the tumor burden, clearly demonstrating the intrinsic killing ability of NK cells in the tumor microenvironment. The considerable decreased expression of stemness markers in residual tumors confirm the CSC-targeting ability of NK cells, in addition to the elimination of differentiated tumor cells. We argue that this tremendous antitumor efficacy is largely related to the extensive tumor infiltration of NK cells, achieved via direct intravesical administration, surpassing the poor infiltration when delivered intravenously (data not shown). This has been observed in animal models and clinical studies, and is considered a critical factor for efficacious adoptive NK cell therapy when delivered systemically [34–36]. The innate ability of NK cells to target both stem and non-stem cell population by NK cells is of utmost importance to achieve a meaningful disease remission and survival benefits since non-stem cells might switch to a stem-like phenotype able to sustain tumor growth.

A limiting factor of this study is the use of immunocompromised mice lacking T cells that play a central role in tumor surveillance [37, 38]. It is well-known that NK cells, through release of cytokines, exchange bidirectional activating signals in a positive feedback with dendritic and CD4^+^ T cells, with consequent enhancement of an antitumor immune response [37, 38]. The lack of this synergistic effect with other immune effectors, only possible in an immunocompetent host, is likely to underestimate the anti-tumor response we have observed in our model, which was nevertheless notably significant. However, future studies using humanized mouse models to reproduce the complex interactions of NK cells with other cells of the immune system are needed to more accurately predict the anti-tumor clinical efficacy of allogeneic NK cell-based immunotherapy.

## Conclusions

We demonstrated, for the first time, that intravesical therapy with ex vivo-activated allogeneic NK cells provides a rapid and noteworthy anti-tumoral response against BC by targeting both stem and non-stem cell populations. Importantly, the ability of NK cells to drive CSC differentiation, viewed as major precursors of muscle-invasive forms, are likely to prevent or delay recurrence and/or disease progression. These findings, although preclinical, provide evidence for the high therapeutic potential of NK cell-based adoptive immunotherapy in the eradication of bladder CSCs, an approach that should be exploited as part of a combinatorial therapeutic strategy in BC.

## Additional files

Additional file 1: Table S1. Primer sequences used in real-time RT-qPCR analysis. (DOCX 16 kb)
Additional file 2: Figure S1. IL-2/IL-15 increases the expression of NK cell-activating receptors. Representative FACS histograms of NK-activating receptors in resting and activated NK cells (gray and white profiles, respectively). Dotted lines represent isotype-matched controls. Bar graphs represent the mean ± SEM (n = 3) of each receptor in fresh and activated NK cells. **P* < 0.05 and ***P* < 0.01 compared to freshly purified NK cells. (TIF 193 kb)

## Abbreviations

ALDH: aldehyde dehydrogenase
BC: bladder cancer
CSCs: cancer stem cells
HD: healthy donors
MIBC: muscle-invasive bladder cancer
NCRs: natural cytotoxicity receptors
NK: natural killer
NMIBC: non-muscle-invasive bladder cancer
