# Supplementary material for: Natural killer cell-based adoptive immunotherapy eradicates and drives differentiation of chemoresistant bladder cancer stem-like cells
Source: BMC Med. 2016 Oct 21;14:163. doi: 10.1186/s12916-016-0715-2 (PMC5075212; doi:10.1186/s12916-016-0715-2)
Supplement: Additional file 1: Table S1. — Primer sequences used in real-time RT-qPCR analysis. (DOCX 16 kb) [file 12916_2016_715_MOESM1_ESM.docx]

**Additional file 1: Table S1.** Primer sequences used in real-time RT-qPCR analysis

| Gene | Primer | Primer Sequence/ENSEMBL Gene ID |
| --- | --- | --- |
| ALDH1A1 | Forward | 5’-TGGCTTATCAGCAGGAGTGT-3’ |
| ALDH1A1 | Reverse | 5’-GCAATTCACCCACACTGTTC-3’ |
| ALDH2 | Forward | 5’-CGAGGTCTTCTGCAACCAG-3’ |
| ALDH2 | Reverse | 5’-GCCTTGTCCACATCTTCCTT-3’ |
| SOX2 | Forward | 5’-CATGCACCGCTACGACG-3’ |
| SOX2 | Reverse | 5’-CGGACTTGACCACCGAAC-3’ |
| ABCB1 | Forward | 5’-GAGGAAGACATCACCAGGTATGC-3’ |
| ABCB1 | Reverse | 5’-GCCAGGCACCAAAATGAAACC-3’ |
| ABCG2 | Forward | 5’-TCAGCGGATACTACAGAGTGTCATC-3’ |
| ABCG2 | Reverse | 5’-ATCTGCCTTTGGCTTCAATCCTAA-3’ |
| HPRT-1 | Forward | 5’-TGACACTGGCAAAACAATG-3’ |
| HPRT-1 | Reverse | 5’-GGCTTATATCCAACACTTCG-3’ |
| 18S | Forward | 5’-GAAGATATGCTCATGTGGTGTTG-3’ |
| 18S | Reverse | 5’-CTTGTACTGGCGTGGATTCTG-3’ |
| GAPDH | Forward | 5’-ACAGTCAGCCGCATCTTC-3’ |
| GAPDH | Reverse | 5’-GCCCAATACGACCAAATCC-3’ |
| CD44 |  | ENST00000278385 |
| CD47 |  | ENST00000398257 |
| KRT14 |  | ENST00000167586 |

All Cycle threshold (Ct) values of the analyzed genes were normalized to the housekeeping genes*, i.e*., HRPT-1, 18S and GAPDH.
