# Supplementary figures and images for: Natural killer cell-based adoptive immunotherapy eradicates and drives differentiation of chemoresistant bladder cancer stem-like cells
Source: BMC Med. 2016 Oct 21;14:163. doi: 10.1186/s12916-016-0715-2 (PMC5075212; doi:10.1186/s12916-016-0715-2)

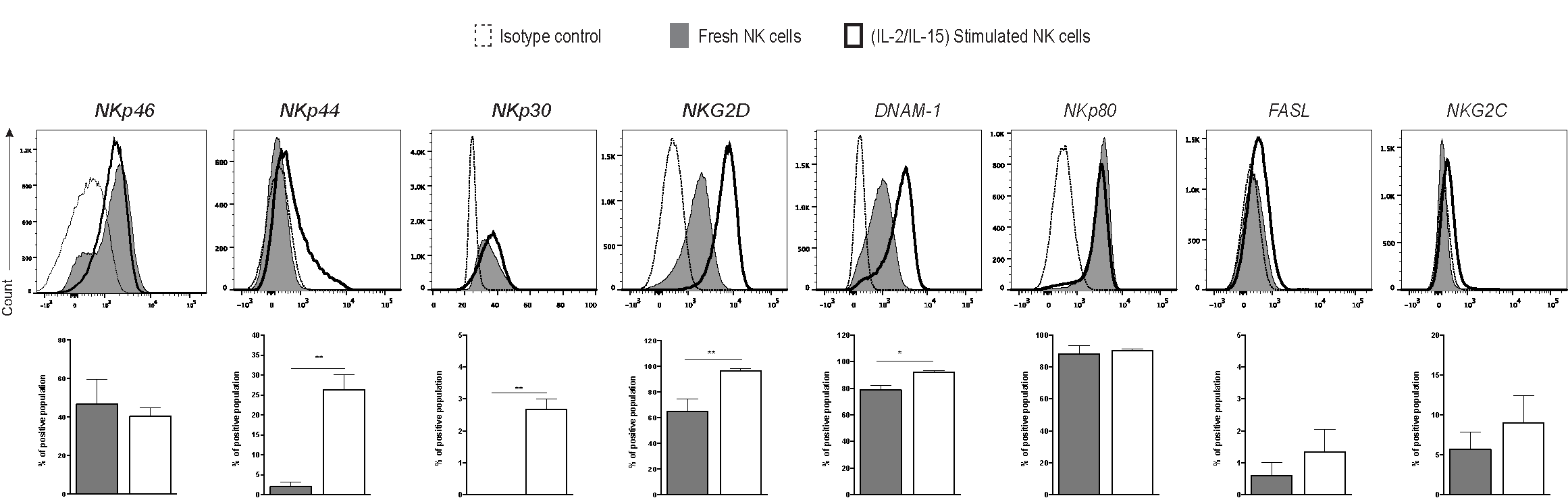

Supplement: Additional file 2: Figure S1. — IL-2/IL-15 increases the expression of NK cell-activating receptors. Representative FACS histograms of NK-activating receptors in resting and activated NK cells (gray and white profiles, respectively). Dotted lines represent isotype-matched controls. Bar graphs represent the mean ± SEM (n = 3) of each receptor in fresh and activated NK cells. *P < 0.05 and **P < 0.01 compared to freshly purified NK cells. (TIF 193 kb) [file 12916_2016_715_MOESM2_ESM.tif]
